# 25-Hydroxycholesterol Effect on Membrane Structure and Mechanical Properties

**DOI:** 10.3390/ijms22052574

**Published:** 2021-03-04

**Authors:** Marco M. Domingues, Bárbara Gomes, Axel Hollmann, Nuno C. Santos

**Affiliations:** 1 Instituto de Medicina Molecular, Faculdade de Medicina, Universidade de Lisboa, 1649-028 Lisbon, Portugal; bgomes@medicina.ulisboa.pt; 2 Centro de Investigación en Biofísica Aplicada y Alimentos (CIBAAL), Universidad Nacional de Santiago Del Estero-CONICET, Santiago del Estero 4206, Argentina; ahollmann@conicet.gov.ar

**Keywords:** cholesterol, 25-hydroxycholesterol, atomic force microscopy, force spectroscopy, supported lipid bilayers

## Abstract

Cholesterol is responsible for the plasticity of plasma membranes and is involved in physiological and pathophysiological responses. Cholesterol homeostasis is regulated by oxysterols, such as 25-hydroxycholesterol. The presence of 25-hydroxycholesterol at the membrane level has been shown to interfere with several viruses’ entry into their target cells. We used atomic force microscopy to assess the effect of 25-hydroxycholesterol on different properties of supported lipid bilayers with controlled lipid compositions. In particular, we showed that 25-hydroxycholesterol inhibits the lipid-condensing effects of cholesterol, rendering the bilayers less rigid. This study indicates that the inclusion of 25-hydroxycholesterol in plasma membranes or the conversion of part of their cholesterol content into 25-hydroxycholesterol leads to morphological alterations of the sphingomyelin (SM)-enriched domains and promotes lipid packing inhomogeneities. These changes culminate in membrane stiffness variations.

## 1. Introduction

Lipid membranes are composed of a plethora of phospholipids, sphingolipids and sterols. Usually, mammalian cell membranes have higher proportions of phospholipids, such as phosphatidylcholine and phosphatidylethanolamine, and lower percentages of sphingolipids and sterols, namely, sphingomyelin (SM) and cholesterol (Chol) [1]. Lipid balance alterations have been linked to several pathological conditions, such as cancer, cardiovascular diseases and diabetes [2]. Hence, the modulation of the lipid membrane composition is considered to be a potential therapeutic target [3].

The specificity of membrane lipid organization guides cellular pathways and functionalities, such as cell polarization and trafficking, signal transduction, cell growth, migration and the entry of viruses, bacteria and nanoparticles [2]. Lipid rafts are membrane-level molecular associations enriched in cholesterol and sphingolipids, involving van der Waals forces and hydrogen bonding [4], which are considered to take part in membrane trafficking and signaling processes [5]. Their presence in lipid mixtures is responsible for changes in membrane properties, with decreased membrane fluidity and higher ordering, creating a liquid-ordered (L_o_) phase in coexistence with a liquid-disordered (L_d_) phase [6,7]. Comparably, when cholesterol is removed from the membrane, packing and rigidity decrease, while water permeability increases [8].

Cholesterol homeostasis is regulated by itself as well as by oxysterols [9]. 25-Hydroxycholesterol (25HC) is more effective in regulating cholesterol biosynthesis than cholesterol alone [10]. 25HC is an oxysterol that presents an additional hydroxyl group at the end of the isooctyl tail of cholesterol. The addition of the hydroxyl group in position 25, in addition to the 3-hydroxyl group, alters the amphiphilic characteristics of this molecule. Studies with 25HC have been conducted to determine its effects on lipid membranes. It was shown to promote the coexistence of liquid phases below the transition temperature in the same way as that described for cholesterol [11]. Furthermore, the interaction of 25HC with phospholipids was associated with a membrane expansion effect, leading to increased bilayer permeability [12,13]. 

Molecular dynamic simulations were also used to evaluate how 25HC interferes with lipid membranes. It was shown that it facilitates membrane bending when compared to cholesterol. Additionally, 25HC adopts a tilted orientation in membranes, with the isooctyl tail bent upwards and the 25-hydroxyl group facing polar head groups [14].

Early studies on 25HC effects were related to atherosclerosis disease progression due to the deregulation of its levels [15,16]. However, the expression of the enzyme responsible for 25HC production, cholesterol-25-hydroxylase (CH25H), by macrophages and dendritic cells elicited the potential effect of 25HC at the immune level [17]. Liu et al. found that both CH25H and 25HC were able to neutralize the replication of enveloped viruses [18]. The presence of 25HC in lipid membranes blocked the HIV-1, vesicular stomatitis and Zika viruses’ entry into their target cells [12]. More recently, it was found that CH25H is induced by SARS-CoV-2 infection in vitro and in COVID-19-infected patients, with different studies reporting the anti-SARS-CoV-2 activity of 25HC [19,20,21]. 

In this work, we used atomic force microscopy (AFM) and AFM-based force spectroscopy to assess the membrane modulation effects of 25HC. The experimental method proposed enables the imaging of the supported lipid bilayer (SLB) structure and the quantification of membrane mechanical resistance, allowing us to evaluate the effects of 25HC in different domains of the same lipid bilayer. 

## 2. Results

### 2.1. 25HC Induces Morphological Changes in the Bilayer Structure

**Figure 1:**
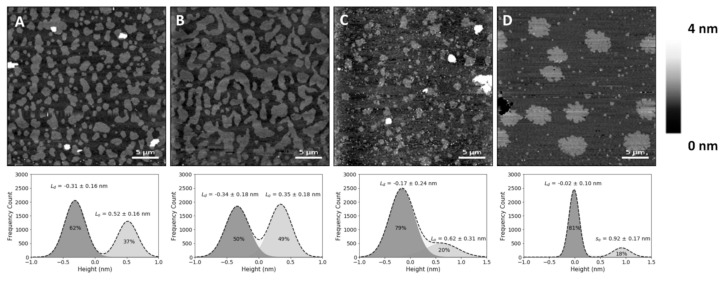
Atomic force microscopy (AFM) height images of supported lipid bilayers. Images of phase separation in binary and ternary lipid bilayers of DOPC:SM:Chol (1,2-dioleoyl-*sn*-glycero-3-phosphocholine:sphingomyelin:cholesterol) or 25HC (top), and respective height differences between the two membrane phases and their area percentage of the whole supported lipid bilayer (SLB), calculated by the fit of a sum of two Gaussians (bottom). (**A**) Bilayer with 20% Chol (DOPC:SM:Chol 2:2:1 in molar ratio, corresponding to 4.32:4.10:1 in molar volume ratios) exhibiting phase separation of liquid-ordered (L_o_) smaller domains. (**B**) Larger-sized L_o_ domains in a phase separation with Chol content increased to 33% (DOPC:SM:Chol 1:1:1 in molar ratio; 2.16:2.05:1 in molar volume ratios). (**C**) Bilayers with 33% 25HC (DOPC:SM:25HC 1:1:1 in molar ratio; 2.16:2.05:1 in molar volume ratios), displaying smaller phase separated domains. (**D**) Bilayer with 50% SM (DOPC:SM 1:1 in molar ratio; 1.05:1 in molar volume ratios) exhibiting a phase separation of solid-ordered (s_o_) domains. All images are 30 × 30 µm^2^.

In the present study, SLBs with equimolar concentrations of 1,2-dioleoyl-*sn*-glycero-3-phosphocholine (DOPC), SM and Chol (corresponding to a molar volume ratio of 2.16:2.05:1, respectively, if one assumes no changes in molar volumes associated with the mixing of these components) yielded differences in height (or thickness) between the L_o_ and L_d_ phases of 0.6 ± 0.1 nm (Figure 1B), which matches previous reports [22,23]. The lighter (higher thickness) domains observed in the SLBs correspond to the SM and Chol-enriched L_o_ phase, coexisting with the darker (lower thickness) background of the DOPC-enriched L_d_ phase (Figure 1A,B). Comparison with the SLBs obtained for the ternary mixture of equimolar concentrations of DOPC, SM and 25HC (Figure 1C) shows differences in height profiles, as well as different phase separations between L_o_ domains and the L_d_ matrix (Figure 1C). The height difference between the L_o_ domains and L_d_ matrix increased to 0.9 ± 0.1 nm.

**Figure 2:**
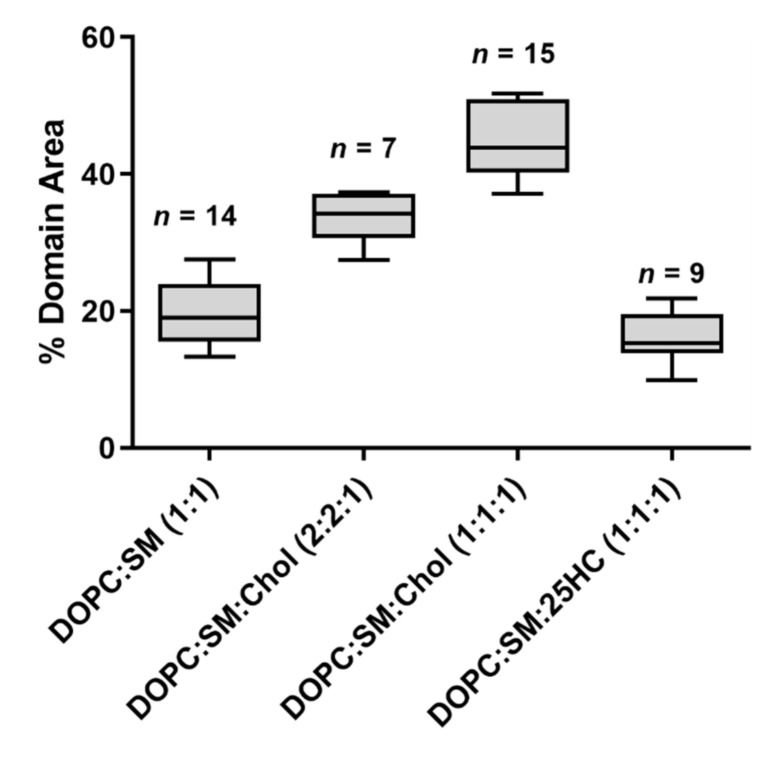
The fraction of the immiscible phase separation area occupancy in the whole SLB, calculated from imaging analysis. Each domain area was calculated from images of 30 × 30 µm^2^. Sample sizes are given above the boxes. All samples passed a normality test. Statistically significant variations were seen for all multiple comparisons of the percentage of domain area (one-way ANOVA and Tukey’s post hoc test, with *p* < 0.05), except for the variation in percentage of domain area between SLBs of DOPC:SM (1:1) and DOPC:SM:25HC (1:1:1), which is not statistically significant (*p* > 0.05).

To evaluate whether the observed changes at the membrane level are 25HC-driven, instead of merely due to the removal of the sterol from the membrane, we studied SLBs composed of an equimolar proportion of DOPC and SM (corresponding to a molar volume ratio of 1.05:1, respectively). These bilayers presented a phase separation with a height difference of 1.0 ± 0.1 nm (Figure 1D) and a fraction of the area occupancy of the thicker domains in the whole membrane of 20 ± 5% (the weighted value obtained from the Gaussian fit is 24 ± 4% for the domain occupancy) (Figure 2). 

**Figure 3:**
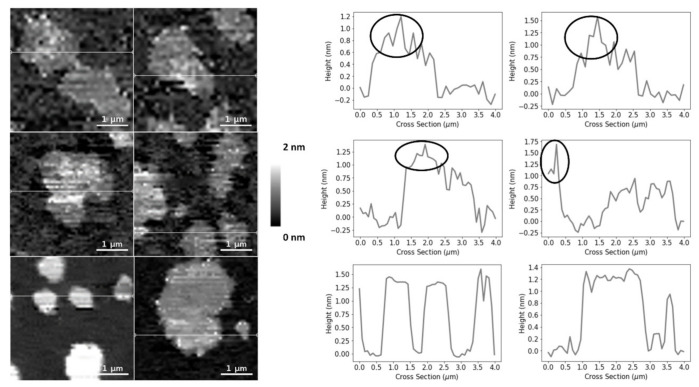
AFM height images and corresponding height cross-sections of SLBs of DOPC:SM:25HC (1:1:1), highlighting the height heterogeneity of the thicker domains. The circles highlight the brighter and thicker SM-enriched areas of the domains. All images are 4 × 4 µm^2^ zooms from the original images of 30 × 30 µm^2^.

The SM and Chol-enriched L_o_ phase in the equimolar DOPC:SM:Chol SLBs occupied 45 ± 5% of the entire membrane area (the weighted value obtained from the Gaussian fit is 51 ± 5% for the domain occupancy). As shown in Figure 1C, the presence of 25HC in the lipid bilayer induced the formation of well-separated smaller domains, when compared to the bilayer of equimolar DOPC and SM (Figure 1D), representing 16 ± 4% of the membrane area (the weighted value obtained from the Gaussian fit is 20 ± 4% for the domain occupancy) (Figure 2). However, imaging shows that cholesterol substitution by 25HC exerts an effect on membrane lipid organization: there is a close resemblance in the topography of bilayers formed of DOPC:SM:25HC (1:1:1) and those of DOPC:SM (1:1). Looking closer at the domains formed in SLBs composed of equimolar proportions of DOPC, SM and 25HC, one may notice that there is heterogeneity in terms of height distribution (Figure 3). We could speculate that smaller quantities of 25HC located in SM-enriched domains may be inserted at higher local concentrations in some regions, inducing a decrease in the height of these regions (Figure 3).

### 2.2. 25HC Induces a Heterogeneous Softning of SM-Enriched Domains

Despite the observation of SLB morphology shed some light on the membrane-level effects of 25HC, it does not reveal quantitative lipid bilayer mechanical properties. Thus, to further evaluate the effects of 25HC, we used AFM-based force spectroscopy to quantify the force necessary to disrupt the lipid bilayer packing.

**Figure 4:**
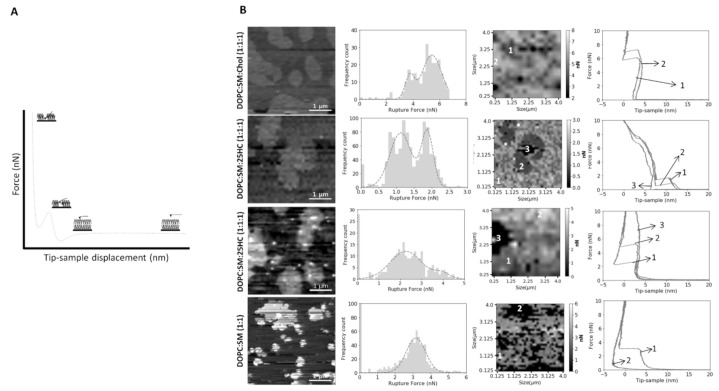
(**A**) Schematic representation of the SLB indentation process using AFM-based force spectroscopy. Typical force distance curve showing the discontinuity in the approach curve when the bilayer is punctured. The different parts of the force curve are represented as steps in the scheme. (**B**) Representative breakthrough force maps, together with the corresponding frequency histograms and force curves, for DOPC:SM:Chol (1:1:1), DOPC:SM:25HC (1:1:1) and DOPC:SM (1:1) bilayers, at a loading rate of 200 nm.s^−1^. There are two images of DOPC:SM:25HC (1:1:1) bilayers for representative data on low- and high-rupture forces on L_o_ domains (numbered as 2), while s_o_ domains are numbered as 3 (or 2 in DOPC:SM SLBs). The L_d_ phase is numbered as 1. All histograms include 1024 or 256 force curves. The gray scale in the force maps represents the force scale in nN. All breakthrough force maps are 4 × 4 µm^2^.

**Figure 5:**
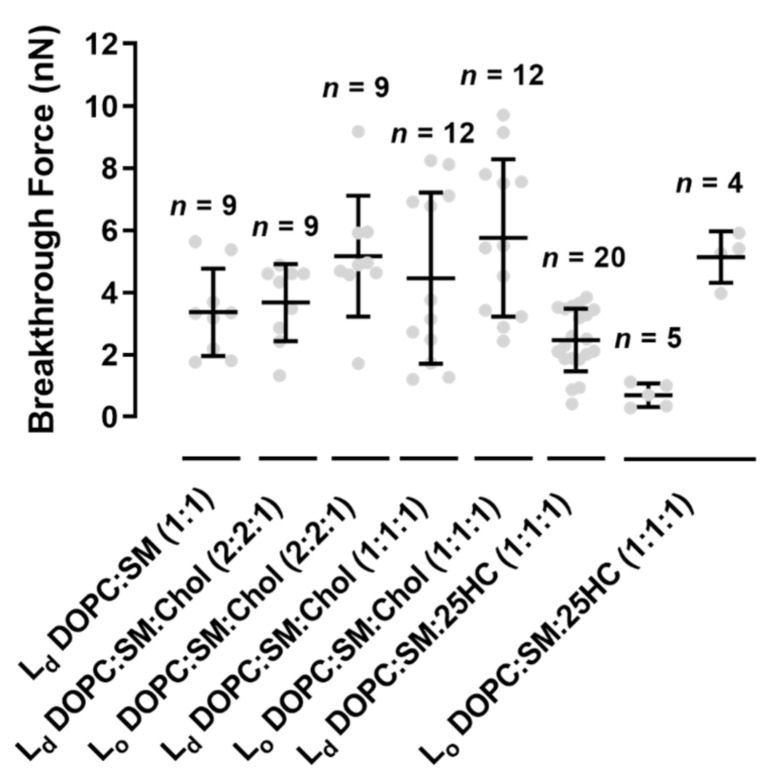
Breakthrough forces for supported lipid bilayers of binary mixtures of DOPC:SM (1:1) and ternary mixtures of DOPC:SM:Chol (2:2:1), DOPC:SM:Chol (1:1:1) and DOPC:SM:25HC (1:1:1), at a loading rate of 200 nm.s^−1^, on liquid-disordered (L_d_) and liquid-ordered (L_o_) membrane domains. Error bars are the mean ± standard deviation (SD) of a set of *n* = 4–20 force maps, each containing 1024 or 256 force curves. Statistically significant differences were seen for L_o_ in DOPC:SM:Chol (2:2:1) and DOPC:SM:Chol (1:1:1) vs. the lowest obtained values in L_o_ of DOPC:SM:25HC (1:1:1) (one-way ANOVA and Tukey’s test, with *p* < 0.05).

We performed force spectroscopy maps on several areas of the SLBs, selected in a low-resolution images of 16 × 16 pixels (total of 256 force curves) or 32 × 32 pixels (1024 force curves), in a membrane area of 4 µm^2^. The breakthrough force is the maximum force load that a bilayer can hold until its rupture, which allows the measurement of the bilayer mechanical stability. Figure 4A presents a typical force curve, as well as the features of the force profile and corresponding parameters measured on the SLBs used in this study, at a loading rate of 200 nm.s^−1^. The lower loading rate allows the softer bilayer sample to be indented by the AFM probe until it cannot hold the force anymore. When the membrane can no longer stand the force applied by the AFM tip, it breaks through the lipid bilayer down to the mica substrate, where it keeps on bending until the selected loading force of 10–15 nN is reached. Figure 4B and Figure 5 show that the L_o_ domains of the ternary mixture of DOPC:SM:Chol (1:1:1) collapse at higher forces than the L_d_ domains (5.8 ± 2.5 nN vs. 4.5 ± 2.8 nN, respectively). The values obtained are near those measured by others at the same loading rate [22].

The replacement of Chol by 25HC in ternary membranes of DOPC:SM:25HC (1:1:1) led to a marked effect on the mechanical properties (Figure 4B). 25HC induced a heterogeneous softness of the L_o_ domains, which is reflected by a higher variability of the breakthrough force values of rupture events in different areas of the domains. In some SLB areas, the presence of 25HC softens the SM-enriched L_o_ domains to lower values, with breakthrough values of 0.7 ± 0.4 nN. These results have a statistically significant variation relative to the breakthrough values obtained for the L_o_ domains of DOPC:SM:Chol (2:2:1) and DOPC:SM:Chol (1:1:1) SLBs: 5.2 ± 1.9 nN and 5.8 ± 2.5 nN, respectively. In other bilayer areas, 25HC incorporation in the domains yielded higher breakthrough forces (5.1 ± 0.8 nN; Figure 4B and Figure 5). Furthermore, in some areas of the SM-enriched domains, no rupture events could be measured. 

To prove that we are evaluating the breakthrough of such SM-rich domains, we conducted the same type of experiments in DOPC:SM (1:1) bilayers (Figure 4B). The breaking of the s_o_ phase of SM-enriched domains was not detectable at the maximum load used in these studies (10–15 nN).

Although no statistically significant differences were found between L_d_ domains of all the bilayers studied, the membranes with 25HC showed a tendency to have decreased breakthrough forces of 2.5 ± 1.0 nN (Figure 5).

**Figure 6:**
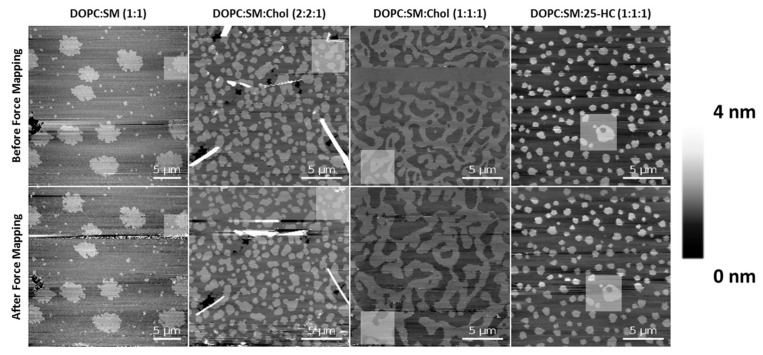
AFM height images before (upper panel) and after (lower panel) force mapping experiments on SLBs of different lipid compositions. The highlighted squares are selected regions of the force mapping.

To rule out possible effects of the tip breakthrough force in these membrane studies, the area of the force mapping was imaged both before and after the indentation experiments (Figure 6). Although some dynamics and changes were observed for the SLBs of DOPC:SM:Chol (1:1:1) after the force mapping experiments, the other compositions essentially retained their morphology and organizational properties of the L_o_ domains after performing those experiments. 

## 3. Discussion

Supported lipid bilayers have been used for studying lipid interactions and membrane properties under different conditions [24,25,26,27,28]. Considering the lack of knowledge on the phase segregation of lipid membranes in the presence of the oxysterol 25HC, AFM imaging was performed to visualize the domain phase separation in SLBs of mixtures of DOPC, SM and Chol or 25HC. Previous studies have demonstrated that increased proportions of Chol cause the coalescence of L_o_ domains [22] enriched in SM and Chol on a continuous background composed mainly of L_d_ DOPC.

As shown in Figure 1, the presence of 25HC induces increased thickness on the phase separation between the continuous matrix and the ordered domains. Olsen et al. studied the perturbations induced by 25HC on the membrane structure [14]. Using molecular dynamics simulations, it was shown that 25HC induces a thinning of the lipid bilayer, with less compacted phospholipids. Another study by Gale et al. showed that 25HC expansion perturbation decreases with increasing phospholipid saturation in the bilayer composition [13]. Thus, the thinning effect of 25HC, mainly located in the unsaturated DOPC phospholipid matrix, may lead to the higher height difference between the L_o_ domains and the L_d_ matrix observed for bilayers containing this oxysterol (Figure 1C).

Regarding the bilayer domain distributions, morphology and occupancy, the presence of 25HC leads to major changes, as shown in Figure 1C and Figure 2.

The SM-enriched domains, for equimolar DOPC and SM SLBs (Figure 1D), present rough edges due to high order packing, which prevents the flow and reordering of the phase boundary into the lowest energy configuration, i.e., close to circular, with smoother edges [24]. On the other hand, the presence of 25HC leads to a transition for less rough edges, as seen for Chol-enriched SLBs (Figure 1A,C).

The bisamphiphilic character of 25HC was shown to lead to a different orientation in the bilayer when compared to Chol [14]. While Chol inserts parallel to the phospholipid tails and induces stronger condensing effects between neighbor phospholipids [29,30], 25HC acts in the opposite way. It was shown that 25HC assumes a highly tilted orientation in bilayers, such that both the 3β- and 25-hydroxy groups interact with the headgroups of phospholipids, leading to stronger expansive effects on the membrane [14,31,32]. This expanding effect may make it more difficult for the L_o_ domains to be formed, in clear contrast with the high proportion of the membrane area corresponding to the L_o_ domain in Chol-containing bilayers (Figure 1A,B and Figure 2). As previously mentioned, Gale et al. demonstrated that the 25HC effect decreases with increasing phospholipid saturation [13]. We infer that a large fraction of 25HC may be located in the DOPC-enriched matrix, with the remaining 25HC distributed in the SM-enriched domains.

Upon analyzing the domains of the DOPC:SM:25HC (1:1:1) bilayers, one can find a heterogeneous height distribution (Figure 3). We could speculate that smaller amounts of 25HC located in SM-enriched domains may be inserted at higher local concentrations in some regions, inducing a decrease in the height of these regions. The thinning effect caused by 25HC may explain the formation of the lower height areas of the domains.

Although there are morphological changes promoted by 25HC on the studied SLBs, we studied the stress resistance of the bilayers to clarify the effects of 25HC on the membrane organization (Figure 4B and Figure 5). The morphological changes and the proposed heterogeneous distribution of 25HC may impose different resistances of the bilayer to AFM tip penetration. In this context, this surface variability could be ascribed to the local concentration of 25HC in each compressed region. Thus, we may hypothesize that the absence of rupture events in some of the force curves measured inside the domains of the bilayers formed by the ternary mixture with 25HC may eventually indicate the presence of rigid SM-enriched solid-ordered phase (s_o_) areas, as seen by others in binary equimolar mixtures of DOPC with SM [33,34]. The absence of force rupture on these SM-enriched s_o_ domains of the DOPC:SM (1:1) bilayers at the maximum payload used in the studies supports our hypothesis (Figure 4B). On the contrary, the areas of the domains where rupture events were detectable should be related to the presence of 25HC in the domains. The 25HC-driven effect of lipid expansion may lead to an easier breakthrough of those particular SM-enriched areas. Another important feature observed is the different force ruptures that occurred in the SM-enriched domains of the DOPC:SM:25HC (1:1:1) bilayers (Figure 4B and Figure 5). In some areas, there was a marked softness of the domains, while in other areas there is a higher mechanical resistance (Figure 4B and Figure 5). This difference may arise from different concentrations of 25HC incorporated into the SM-enriched domains, leading to different lipid packing and affecting the way the bilayer can hold the pressure of the tip indentation before breaking. On the other hand, no heterogeneity was observed in the L_d_ matrix of DOPC:SM:25HC (1:1:1) mixtures, suggesting a homogenous distribution of 25HC in this phase. The tendency towards lower values of breakthrough force of the L_d_ matrix on 25HC bilayers, when compared to the L_d_ matrices of the other studied bilayers (Figure 5), suggests that 25HC incorporation into the DOPC-rich matrix leads to a lower packing and higher perturbation of the phospholipid organization, as proposed in previous studies [13,14,31,32].

## 4. Materials and Methods

### 4.1. Materials

1,2-Dioleoyl-*sn*-glycero-3-phosphocholine (DOPC), egg sphingomyelin (SM) and 25-hydroxycholesterol (25HC) were purchased from Avanti Polar Lipids (Alabaster, AL, USA). Cholesterol (Chol) was from Sigma-Aldrich (St. Louis, MO, USA). The working buffer used throughout the studies was HEPES 10 mM pH7.4 in NaCl 150 mM, prepared using Milli-Q water, unless otherwise stated.

### 4.2. Supported Lipid Bilayer Preparation

Phospholipids and Chol were dissolved in chloroform in 5 mM stock solutions. 25HC was dissolved in ethanol, yielding 24.8 mM stock solutions. SLBs were formed by the vesicle fusion rupture method [35,36]. Lipid solutions were mixed at the appropriate molar proportions in a round bottom flask, dried under a nitrogen stream and left overnight in vacuum to remove any traces of solvent. The lipid films were hydrated in buffer for imaging measurements or in Milli-Q water for breakthrough force measurements, to a lipid concentration of 1 mM. The lipid mixtures studied were DOPC:SM:Chol (2:2:1 in molar ratio; 4.32:4.10:1 in molar volume ratio, assuming no changes in molar volume associated to the mixing of the lipids), DOPC:SM:Chol (1:1:1 in molar ratio; 2.16:2.05:1 in molar volume ratio), DOPC:SM:25HC (1:1:1 in molar ratio; 2.16:2.05:1 in molar volume ratio) and DOPC:SM (1:1 in molar ratio; 1.05:1 in molar volume ratio). The multilamellar lipid suspension was power sonicated using a Vibra-Cell ultrasonicator (Sonics & Materials, Newtown, CT, USA) 3 times, in cycles of 3 min with pulsed sonication and 3 min of resting in ice. The clear lipid suspension was centrifuged in a microcentrifuge Z 233 M-2 (HERMLE Labortechnik, Wehingen, Germany) for 5 min at 16,500× *g* to remove titanium particles, large vesicles and debris. 

After this, 500 µL of 10× diluted lipid suspension was pipetted onto freshly cleaved mica along with a 3 mM final concentration of CaCl_2_ in a custom-built well. The sample was incubated in a humidity chamber at 60 °C, above all lipids’ main transition temperature, for 40 min. This procedure allows small unilamellar vesicles (SUVs) to adsorb and rupture on the surface of the mica, forming a flat continuous bilayer [35,36].

After incubation, the bilayer was washed 10–25 times with 100 µL of warm (60 °C) HEPES buffer or Milli-Q water using a pipette. The washing procedure was performed parallel to the bilayer surface. This ensures that unfused vesicles, either in suspension or deposited on the bilayer surface, are removed. In all samples, the hydrated bilayers were cooled down at room temperature, enabling phase separation to occur.

### 4.3. Atomic Force Microscopy Imaging and Force Mapping

Atomic force microscopy was performed using a JPK Nanowizard IV (JPK Instruments, Berlin, Germany). Bilayers were imaged in contact mode and quantitative imaging (QI) mode, a recent innovation in which the apparatus modulates the z-piezo to perform a fast force curve on each pixel of the image [37,38]. This avoids lateral friction and allows for better control of the tip force during measurements. The QI mode allows several mechanical properties to be calculated from the force applied and the tip-sample separation. Throughout the imaging, the maximum applied force was 200 pN, in order not to affect the sample structure [24]. Images were obtained with a resolution of 256 × 256 pixels, at a scan rate of 1 Hz. AFM measurements were performed at room temperature, which varied from 22 to 25 °C.

Before measurements, cantilever spring constants were quantified by the thermal noise method [39], and cantilever sensitivity was measured by performing a force curve on a clean, freshly cleaved mica surface in HEPES buffer or in Milli-Q water. OMCL-TR400PSA AFM probes (spring constant: 0.13 ± 0.01 N/m; sensitivity: 12.75 ± 1.39 nm/V) were used for imaging in contact mode, while qpBioAC CB2 probes (spring constant: 0.06–0.18 N/m; sensitivity: 7.6 ± 1.2 nm/V) were used in QI mode. Approximately 3–5 non-contiguous areas of 10 μm^2^ and 20 μm^2^ from at least 3 bilayers, prepared on different days, from different lipid stocks, were imaged to obtain representative data and assure the reproducibility of the measurements.

Topographical images were analyzed with first or second level flattening, using the JPK data-processing software. A custom written code in Python was used to obtain the image’s height difference between the immiscible and fluid phases and their domain area ratios on the whole SLB. Open source ImageJ software codes were used for imaging analysis to obtain quantitative data of domain areas and corroborate the weights of the domain area ratio calculated from the Gaussian fit.

Force mapping experiments were conducted over 4 μm^2^ (16 × 16 or 32 × 32 pixels) bilayer areas using the same OMCL-TR400PSA AFM probes. Typically, an applied load of up to 10–15 nN and a piezo loading rate of 200 nm.s^−1^ were used. The collected force curves were batch-analyzed using a self-developed Python program. Briefly, for each approach curve, the program looks for a negative difference between two consecutive force values within a range of 1–4 nm piezo displacement. Upon finding this negative difference, the program stores the force value corresponding to the approach curve of that pixel. A force map is then built based on the measured breakthrough force for each pixel.

## 5. Conclusions

In summary, we show that 25HC modulates the membrane structure by reverting the lipid condensing effects typical of the presence of cholesterol, rendering the bilayers less rigid. Furthermore, the distribution of 25HC in lipid membranes seems dispersed in both liquid-disordered and liquid-ordered phases. By the methodologies applied in this study, we could dissect the effect of 25HC in each phase. The structural effects of 25HC observed on lipid membranes should be translated with caution to biological systems. Several physiological mechanisms require cholesterol-induced lipid condensing effects, the so-called lipid raft formation, for receptor clustering, such as T-cell receptor clustering and/or signaling activation pathways [40]. From the perspective of pathophysiological mechanisms, enveloped viruses require fusion between the viral membrane and the cell (or endosomal) membrane to infect a target cell. Virus surface proteins mediate the process by interacting with receptors located (at least in some viruses) in cholesterol-enriched areas of the host cell membrane [41]. The disruption of cholesterol-mediated lipid condensed domains by 25HC may pose an alternative to inhibit viral entry and infectivity, as well as to modulate overexpressed signaling or trafficking in diseases, and may be part of the molecular-level explanation of the mechanism of 25HC antiviral action [12,42].

## Data Availability

Not applicable.
